# Diabetic Retinopathy: Mechanism, Diagnosis, Prevention, and Treatment

**DOI:** 10.1155/2015/854593

**Published:** 2015-03-19

**Authors:** Mohamed Al-Shabrawey, Wenbo Zhang, Denise McDonald

**Affiliations:** ^1^Oral Biology and Anatomy, Ophthalmology and Culver Vision Discovery Institute, College of Dental Medicine and Medical College of Georgia, Georgia Regents University, Augusta, GA 30912, USA; ^2^Department of Ophthalmology & Visual Sciences, University of Texas Medical Branch, Galveston, TX 77555, USA; ^3^Centre for Experimental Medicine, School of Medicine, Dentistry and Biomedical Sciences, Queen's University Belfast, Institute of Clinical Sciences, Block A, Grosvenor Road, Belfast BT12 6BA, UK

Diabetic retinopathy (DR) is the most common complication of diabetes and remains a major cause of preventable blindness. Anatomical and functional changes occur in various retinal cells including retinal endothelial cells, neurons, and retinal pigment epithelium prior to clinical symptoms of the disease. Early changes include appearance of microaneurysms, leukocyte adhesion, apoptosis of vascular (endothelial cells and pericytes), and neuronal cells. The changes progress to involve breakdown of the inner and outer blood retinal barriers causing diabetic macular edema, the most leading cause of vision loss in DR. Capillary degeneration and development of acellular capillaries lead to impairment of retinal perfusion and subsequent hypoxia and retinal neovascularization, the hallmark of proliferative diabetic retinopathy (PDR). There are several therapeutic strategies to manage the DR including laser photocoagulation, anti-VEGF, and triamcinolone intraocular injection. These therapeutic interventions are still limited by significant side effects. Therefore, there is still an urgent need to find out new therapies to limit the diminution or loss of vision in diabetic patients. The current special issue through a number of investigators and experts in the field of DR presents both research and review articles that highlight novel pathways implicated in the development of DR and review the pathophysiology and management of DR.

*Novel Mechanisms of Diabetic Retinopathy.* Effective therapeutic approaches to restore sight in diabetic patients with clinically identifiable retinopathy are still lacking and in this issue S. Z. Safi et al. provide a timely review of current thinking in field. Firstly, using several scientific databases (PubMed, Ovid MEDLINE, SPORTDiscus, and EMBASE databases) they have reviewed the literature focusing on the molecular mechanisms involved in the pathogenesis of DR and, secondly on emerging strategies under consideration for development of future pharmacological interventions. Initially, they described the major pathways widely recognized to be involved in disease, namely, the polyol pathway, activation of protein kinase C (KPC) isoforms, increased hexosamine pathway flux and increased advanced glycation end-product (AGE) formation, and oxidative stress along with several other mechanisms such as the potential role of the renin-angiotensin system which are less well researched. Arising from this discussion they summarized established preventive measures including general, primary, and secondary preventive strategies before covering novel and emerging therapeutic targets such as PKC inhibitors, VEGF inhibitors, and ACE inhibitors and drugs such as antioxidants. Finally, the authors draw on current evidence and clinical studies arguing for the use of fenofibrate in halting disease progression. This review, therefore, provides background and context for the research articles included in this special issue.

Reactive oxygen species (ROS) generated from mitochondria, NADPH oxidase, and other oxidases are known to play an essential role in the pathogenesis of DR ROS modify redox sensitive kinases and transcription factors such as NF-*κ*B, Signal Transducers and Activators of Transcription proteins (STAT), and activator protein 1 (AP-1) and therefore induce inflammatory gene expression in DR. Alternatively, lipid peroxidation and DNA damage induced by ROS cause cell death in DR. In this issue, R. Liu et al. discovered an alternative mechanism on how ROS may play a role in DR. Using human endothelial cells (ECs), they found that ROS treatment induces EC senescence. With gain-and-loss function approaches, they provided the first evidence that ROS-induced EC senescence is at least partially mediated by downregulation of Sirtuin 6 (Sirt6), H3K9, and H3K56 deacetylase recently recognized as a novel antiaging and anti-inflammatory molecule. Further, they found that Sirt6 antagonizes ROS-induced activation of senescence pathways, such as p21 overexpression and activation of retinoblastoma (Rb) protein. As accelerated aging is currently recognized as one of the key mechanisms of many cardiovascular diseases including DR, this study may lead to a hot area to explore Sirtuins as well as other antiaging molecules in DR. Certainly, the role of Sirt6 in diabetic animals and patients remains to be further elucidated. Increased H3K9 acetylation has been found in DR as well as other inflammatory conditions, suggesting that loss of Sirt6 protein and/or function is very likely involved in DR. A challenge to directly address the role of Sirt6 in DR is the lack of transgenic animals. Global Sirt6 knockout mice die within 1 month and EC-specific Sirt6 knockout mice will be important to test whether lack of Sirt6 will accelerate diabetes-induced retinal vascular alterations such as permeability and capillary degeneration if these mice are viable.

There is now much evidence to indicate the involvement of a dysregulated inflammatory response in early disease and defining this role further is likely to offer exciting new avenues for therapeutic intervention. To this end, S. Fulzele et al. have focused on characterizing the regulation of adenosine deaminase 2 (ADA2) by miR-146-3p. They show that the predicted miR-146-3p target site in the UTR of ADA2 is indeed functional. In addition, since the expression of this microRNA is inversely related to ADA2 levels in samples from diabetic patients and similarly treated in vitro modes the authors conclude that miR-146-3p has a regulatory role in diabetes related inflammation.

Most of the research on the pathogenesis of DR has primarily focused on the injury of the neuroretina and the dysfunction of the inner blood retinal barrier (BRB). Contrary, the impact of diabetes on the function of retina pigment epithelium (RPE) has received less attention and also the underlying mechanism for RPE dysfunction during diabetes remains unclear. The paper by S. Beasley et al. demonstrated for the first time the important role of caspase-14 in the development of diabetic macular edema (DME). The expression of caspase-14 in cultured retinal pigment epithelial cells (ARPE-19) is correlated with the disruption of RPE barrier function and hyperpermeability. In addition, knocking down the caspase-14 in RPE abrogated the hyperglycemia-induced RPE hyperpermeability. Interestingly, this paper also demonstrated that caspase-14 might play a role upstream from other caspases to mediate inflammatory and apoptotic responses. In conclusion, this paper suggests caspase-14 as a novel player as well as therapeutic target in DR.

*Biomarkers of Diabetic Retinopathy.* Diabetic retinopathy is a progressive disease, which is clinically identifiable only at an advanced stage; therefore markers that indicate early disease status would be of major benefit in managing disease progression. Since there are early subclinical changes occurring in retina prior to clinical symptoms, it is necessary to find out specific early biomarkers that predict the pattern and progress of these changes to an advanced stage of DR. The discovery of biomarkers to aid in the identification of patients most likely to develop severe DME and PDR is essential for better treatment of this disease. In support of this concept, B. A. Mysona et al. presented here an interesting clinical study in which they tested whether changes in proNGF/NGF levels observed in vitreous will be matched in serum and thus provide rationale to examine proNGF as a biomarker for DR. This study included analysis of serum and vitreous samples from nondiabetic patients and patients with PDR. Interestingly, the proNGF/NGF imbalance in serum was comparable to the imbalance of proNGF/NGF in vitreous of patients with PDR suggesting that serum proNGF/NGF ratio might act as a novel biomarker that reflects the progress of DR.

Increasing evidence indicates that inflammation is a key player in DR. Increases in vitreous inflammatory cytokines such as IL-6, VEGF, MCP-1, and IP-10 have been found to be positively associated with the progression of DR and the severity of macular edema. However, it is difficult to get vitreous samples and therefore the feasibility of using vitreous cytokines as biomarker is very low. In this issue, N. Dong et al. analyzed an array of inflammatory cytokines in the aqueous humor in relation to macular edema in diabetic patients following uncomplicated phacoemulsification cataract surgery. They found that concentrations of inflammatory cytokines such as IL-1*β*, IL-6, IL-8, MCP-1, IP-10, and VEGF are positively associated with macular edema whereas levels of anti-inflammatory cytokines such as IL-10 and IL-12 are negatively associated with macular edema. Given that it is easy to obtain the aqueous humor, this study highlights the possibility to use these cytokines as biomarkers for diabetic macular edema. Certainly, it is much easier to obtain tears than aqueous humor; it would be interesting to further explore whether there is an association of tear inflammatory cytokines with diabetic macular edema and neovascularization. This study also brings an interesting question about where these inflammatory cytokines are generated. Are they diffusing into aqueous humor or are they generated from cells in ciliary body or cornea? Further exploration of their source will help to better understand the development of DR.

*Mohamed Al-Shabrawey* *Wenbo Zhang* *Denise McDonald*

